# Study of Microstructure and Mechanical Properties Effects on Workpiece Quality in Sheet Metal Extrusion Process

**DOI:** 10.1155/2015/848126

**Published:** 2015-07-02

**Authors:** Chatkaew Suriyapha, Bopit Bubphachot, Sampan Rittidech

**Affiliations:** Heat Pipe and Thermal Tools Design Research Unit (HTDR), Faculty of Engineering, Mahasarakham University, Kantharawichai District, Mahasarakham 44150, Thailand

## Abstract

Sheet metal extrusion is a metal forming process in which the movement of a punch penetrates a sheet metal surface and it flows through a die orifice; the extruded parts can be deflected to have an extrusion cavity and protrusion on the opposite side. Therefore, this process results in a narrow region of highly localized plastic deformation due to the formation and microstructure effect on the work piece. This research investigated the characteristics of the material-flow behavior during the formation and its effect on the microstructure of the extruded sheet metal using the finite element method (FEM). The actual parts and FEM simulation model were developed using a blank material made from AISI-1045 steel with a thickness of 5 mm; the material's behavior was determined subject to the punch penetration depths of 20%, 40%, 60%, and 80% of the sheet thickness. The results indicated the formation and microstructure effects on the sheet metal extrusion parts and defects. Namely, when increasing penetration, narrowing the die orifice the material flows through, the material was formed by extruding, and defects were visibility, and the microstructure of the material's grains' size was flat and very fine. Extrusion defects were not found in the control material flow. The region of highly localized plastic deformation affected the material gain and mechanical properties. The FEM simulation results agreed with the experimental results. Moreover, FEM could be investigated as a tool to decrease the cost and time in trial and error procedures.

## 1. Introduction

**Figure 1:**
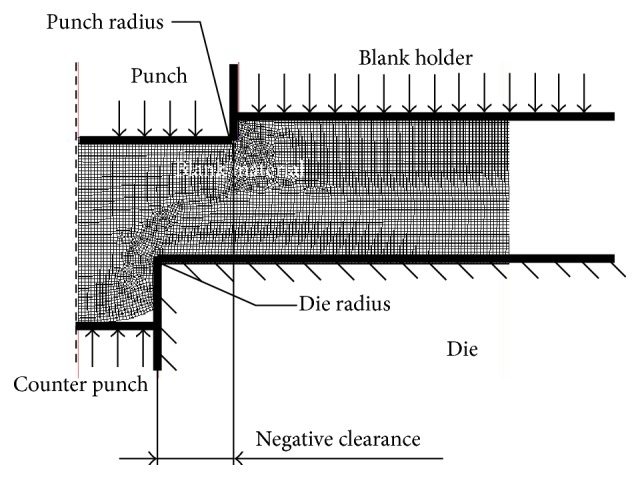
Configuration of sheet metal extrusion modeling.

The process of forming with sheet metal extrusion (SME) is achieved with a punch that has a diameter which is larger than the die hole; that is, there is a negative clearance [1, 2], as shown in Figure 1. Therefore, SME is applied in many industrial fields to form accessories that require 3D parts, small composition, and micro components [3–5]. The advantages of the SME process are that it is possible to produce accessory parts with protruding areas and a blind cavity on the sheet. Many researchers have focused on this field to overcome the associated problems and to find the best solutions for them. Zhuang et al. [3] analyzed the SME process using a finite element method (FEM). Hirota [5] fabricated a microbillet by sheet extrusion, while Hirota et al. [6] produced an experimental and numerical study on a blanking process with negative clearance. Chen et al. [7] simulated the SME process by the enhanced assumed strain FEM.

Although the results of most of the previous studies agreed with the theoretical prediction of the formation of SME parts, there was no explanation of the material behavior that took into account the thickness and microstructure of the SME parts.

This study firstly identifies the geometry and microstructure that would be expected from an experiment. Next, the FEM was used to perform an analysis of the material flow and to explore the influence on punch penetration of the geometry and microstructure. The FEM results were compared with the experimental result to verify the accuracy of the analysis.

## 2. Materials and Methods

### 2.1. Experiment Method

**Figure 2:**
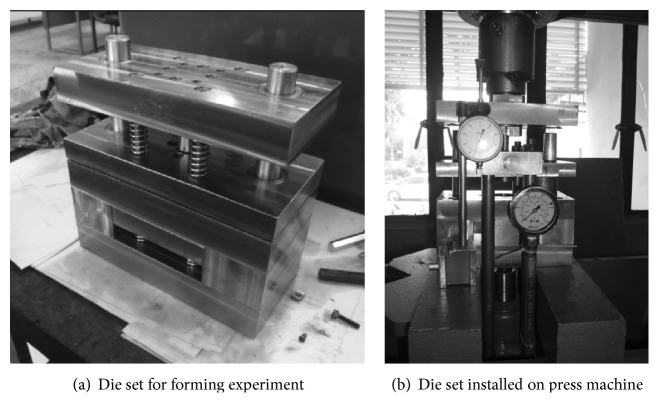
Equipment and tools used in experiment.

Carbon steel is used for a diverse range of applications within a variety of different fields, such as the structural industry, the automotive industry, and the electronics industry, for example [8, 9]. Therefore, the material specimen employed in this study was AISI-1045 steel with a sheet thickness of 5 mm and blank diameter of 38 mm. The punch and die diameters were 11.27 mm and 10.00 mm, respectively, and were formed via a SME process with extrusion ratio *R* = 1.27.  Figure 2 shows the material and specimen used in the experiment: AISI-M4 high speed tool steel was used as the punch, die, blank holder, and counter punch. Forming oil was used for forming lubrication.

The blank holder and counter punch had the lowest force possible applied in the SME process because the study aimed to observe the material flow behavior effect on each tool, and the forces were 16 kN and 500 N, respectively. The visible etching under an optical microscope (50x) was the initial observation made to study the material flow direction and characteristics of the microstructure. The FEM simulation will be used as an important tool for the study of the behavior of material flow, principal stress maximum, and principal plastic strain maximum on the AISI-1045 steel formation and microstructure effect in the SME process.

### 2.2. FEM Simulation Method

To achieve this research, the FEM simulation approach will be used to explore the capabilities of the SME. The SME process was simulated using a 2D axisymmetric model, in which only half of the part was modeled, as shown in Figure 1. In this study, the punch, die, guide ring, blank holder, and counter punch were considered to be rigid models.

**Figure 3:**
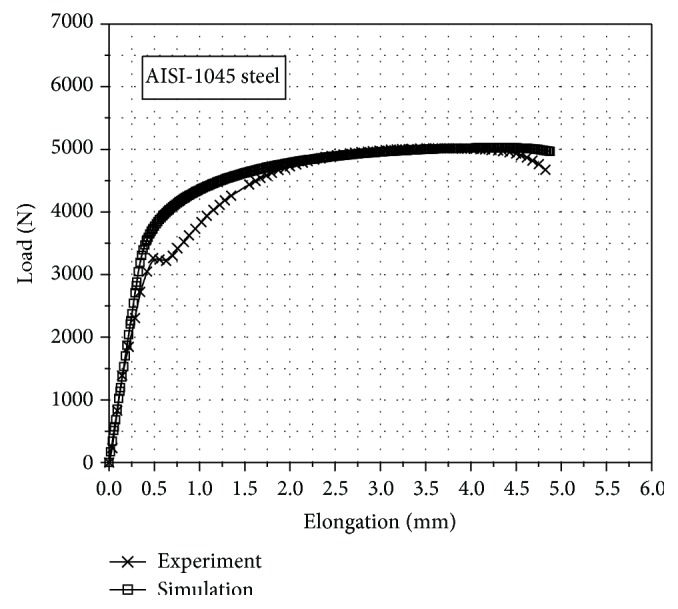
Comparison of tensile test between experimental and FEM simulation results.

The FEM equation was primarily verified from tension simulation and tension test results. AISI-1045 steel was used as the blanked material, the flow curve equation and mechanical properties were determined by tensile test, and the coefficient of friction was determined by ring compression. In Figure 3 the FEM results of the tensile test show that the elongation was 26% and the tensile strength was 530 MPa, which had errors of approximately 0.83% and 2.05% compared with the tension test experiment results. The friction coefficient for the FEM simulation was obtained by employing the ring compression test, and it was 0.04 [10, 11].

Table 1 shows the flow curve equation that was determined form the tensile testing results. Therefore, the strength coefficient value was 850 and the strain hardening exponent value was 0.478. The critical fracture value and fracture criterion equation were considered in this study from the forming investigation of the FEM simulation. The fracture criterion equation presented by Oyane et al. [12] was used, where the constant *α* was 1 and the critical fracture value *C* was 0.157. The validated confirmation of the FEM simulation results obtained using each set of conditions and equations was verified for the analysis of the formation and microstructure effect on the SME process.

## 3. Results

### 3.1. Confirmation of FEM Simulation Equations

**Figure 4:**
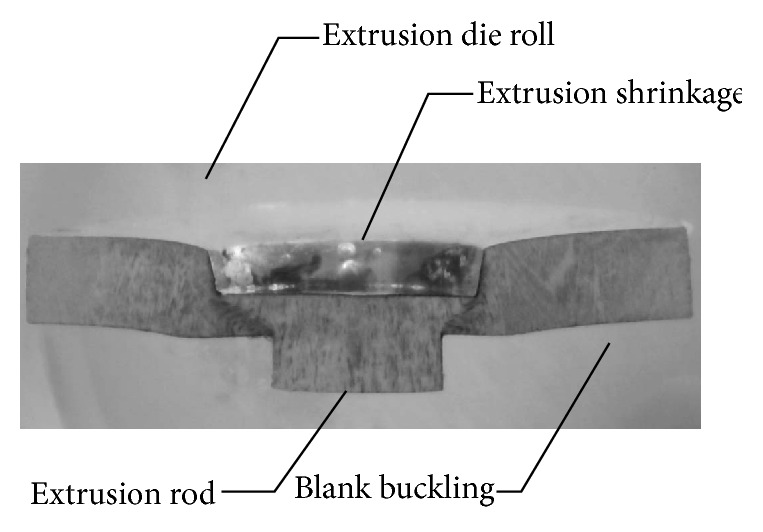
Cross section part of SME configuration.

In this study, the experimental result confirmed that the part's geometry measurement values between the actual forming and simulation results were the same. In the SME experiment, the punch element was able to penetrate gradually the blank to a thickness of about 80 percent, as shown in Figure 4. The blank material was pushed into the extrusion hole by the extrusion rod. Moreover, the deflections in the SME piece appeared, and they were consistent with the extrusion die roll, extrusion shrinkage, and blank buckling. However, die roll defects become visible around the extrusion cavity: extrusion shrinkage was caused by the extrusion rod and blank buckling was caused by the punch's travel.

Thus, the size and geometric figure measurement values between the actual part and FEM simulation results were confirmed as being the same. The geometry measurement considered four points: (I) depth of extrusion die roll, (II) depth of extrusion shrinkage, (III) blank buckling space, and (IV) length of extrusion rod.

**Figure 5:**
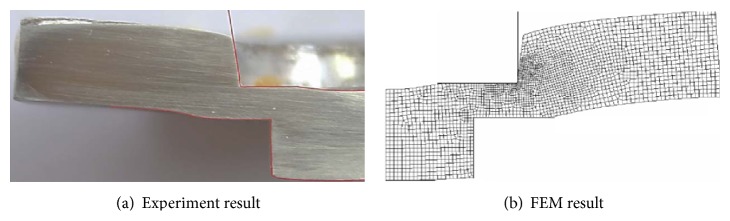
Comparison between actual part and FEM simulation.

**Figure 6:**
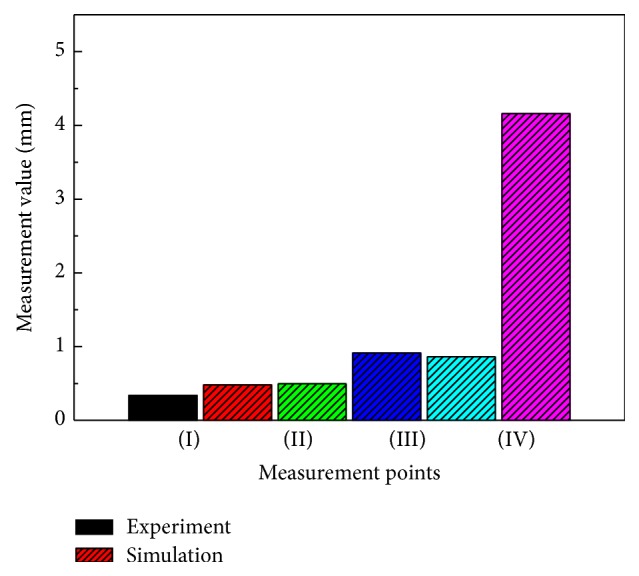
Comparison of experimental and FEM.

Figure 5 shows a comparison between the actual part and FEM simulation. The SME geometry of the experimental and FEM results had identical aspects. Namely, both results were identical in the aspect sizes, geometric figure and formation defects, as shown in Figure 5, and the comparison of measurement results in Figure 6.

The results of the comparison of the experimental and FEM measurement values are shown in Figure 6; the errors of the geometric sizes between the FEM and experimental results for the depth of extrusion die roll, depth of extrusion shrinkage, blank buckling space, and the length of extrusion rod are 3.43, 3.54, 5.39, and 6.65 percent, respectively, and the average error was 4.75 percent. Therefore, the geometric size of the FEM model affected the FEM accuracy results. Namely, the FEM results had slight errors in the small parts and much larger errors in large parts measured.

### 3.2. Comparison of Formation Analysis between Experimental and FEM Simulation Results

**Figure 7:**
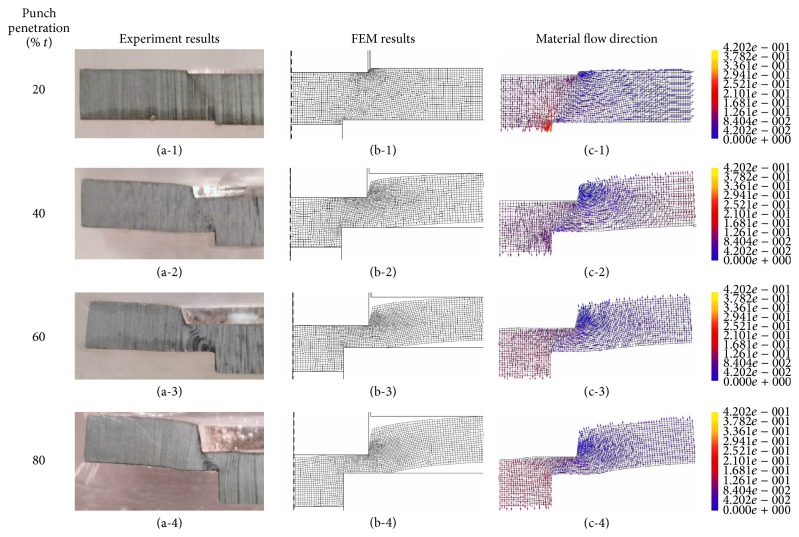
Comparison of formation behavior analysis between experimental and FEM simulation results.

In this study, the punch traveled gradually to penetrate the blank material, and this was used to investigate the SME formation under four sheet thicknesses of 20%, 40%, 60%, and 80%. In Figure 7(a-1), the punch penetrates 20% of the blank thickness, and the extruded part was deflated in the extrusion cavity; protrudes on the opposite side and some material were moved between the punch and die edge. The velocity range was the levels adjusted so that the material flow features near the formation area on the material blank were clear. The more the material was deformed near the tooling edges, the higher the velocity flow around the die hole is, which meant that some material could not flow into the die orifice so there was lateral flow along the die surface as in Figures 7(b-1) and 7(c-1).

The more the punch penetrated the blank material, the more material that flowed through the orifice was limited, and more material flowed laterally due to greater resistance from the blank holder that affected the die roll, while blank buckling appeared clearly on the piece part, as shown in Figures 7(a-2), 7(b-2), and 7(c-2).

The material flow continued during the SME process; some material was compressed and did not flow into the die orifice and there was nonlateral flow along the die surface that caused a dead metal zone near the region of cutting between the punch and die edges. The material near the punch edge had lateral flow and the blank holder resisted, which caused the large die roll, blank buckling to be greater and the twist on the blank end. The surface of the extrusion rod was in contact with the orifice die surface, and the friction caused the velocity of the material flow to be lower than the extrusion core center; the extrusion rod then begins to appear rounded, as shown in Figures 7(a-3), 7(b-3), and 7(c-3).

In Figure 7, the 80% blank thickness was the final one formed, and the dead metal zone was subject to intense compression, with the material having difficulty flowing into the nearest dead metal area. The material near the punch surface was internal sheared to flow past the dead metal region into the die orifice.

Therefore, the material behavior as above has the extrusion shrinkage appearance. Furthermore, there was internally sheared material that passed the dead metal region laterally and became more evident on the blank buckling and extrusion die roll.

### 3.3. Comparison of Microstructure Behavior Analysis between Experimental and FEM Simulation Results

**Figure 8:**
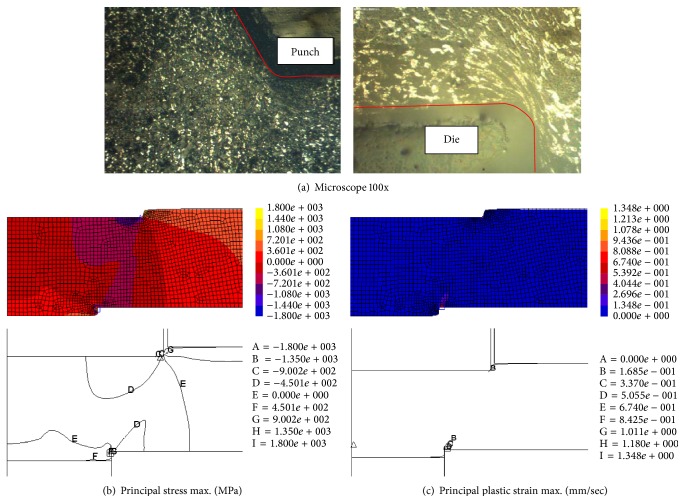
Microstructure behavior analysis for 20% punch penetration.

In the earliest stage of the SME process, as shown in Figure 8, the material protruded trough the die orifice due to the thrust of the punch. The material grain was slightly flat near the punch edge, and the material compressive reaction, as the compression stress, mainly appeared around the punch edge. In contrast, the material nearby the die edge was pushed forward easily, and tension strain was mostly present in such an area, so the material grain was flatter.

**Figure 9:**
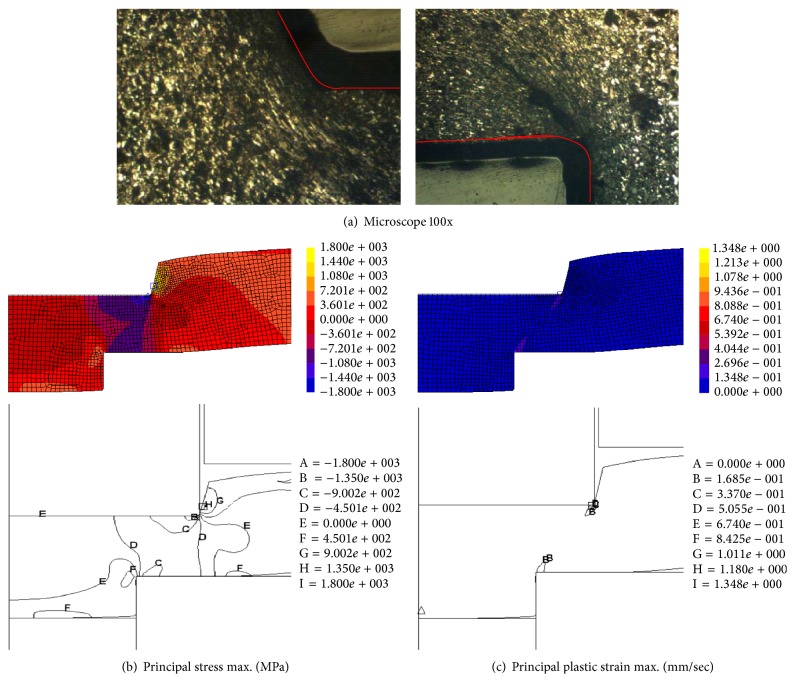
Microstructure behavior analysis for 40% punch penetration.

Figure 9 shows that when the thrust punch penetrated 40% of the blank's thickness, the material's grain was increasingly flat around the forming tool edges; the compression stress and strain were intense around the punch edge and suffused between the punch and die edges. The material thrust flow caused the blank to bend and die roll defects were visible.

**Figure 10:**
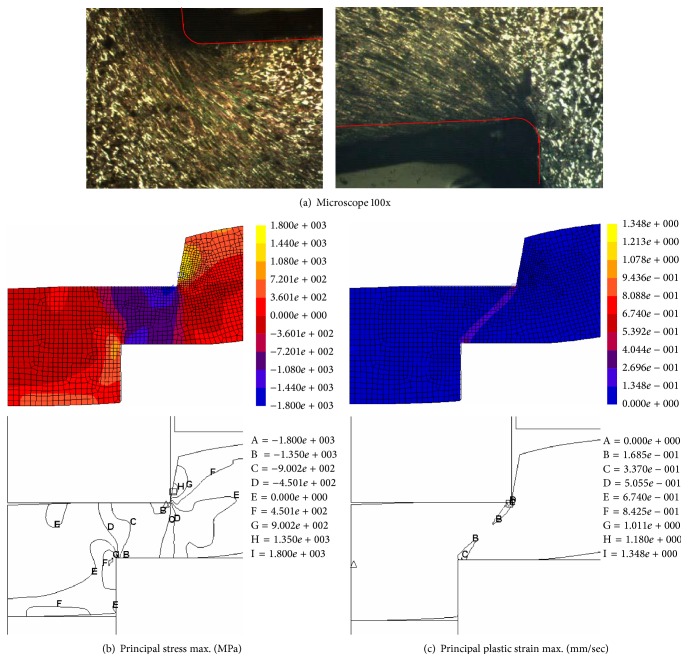
Microstructure behavior analysis for 60% punch penetration.

For the thrust punch that penetrated 60% of the blank's thickness, as shown in Figure 10, the material's grain was increasingly flat and pervaded around the forming tool edges; the compression stress was intense and expanded in the blank material. The compressive strain was intense around the punch edge and there was increased convergence between the punch and die edges.

**Figure 11:**
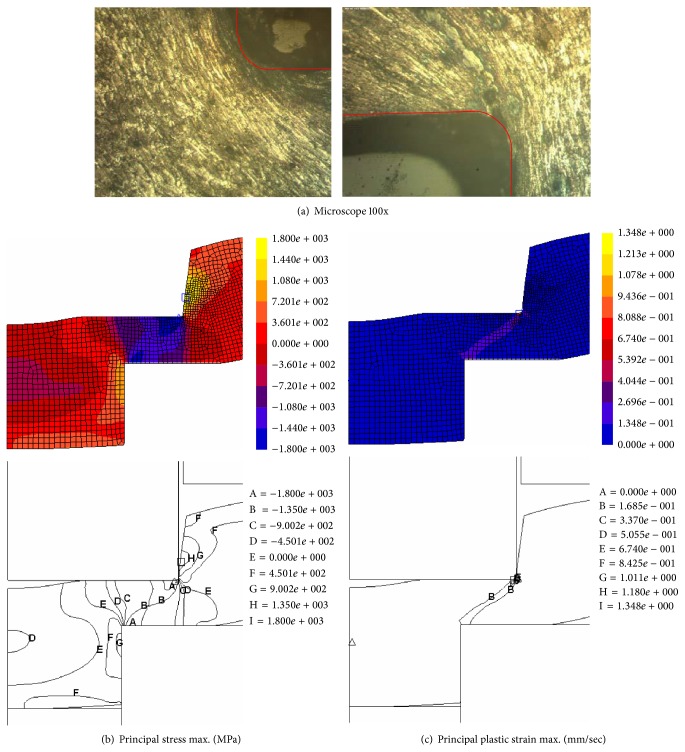
Microstructure behavior analysis for 80% punch penetration.

The final state of the SME process experiments and FEM simulation was a thrust punch that penetrated 80% of the blank's thickness, the material's grain was extremely fine and pervaded the forming tools' surface, and the compression stress intensity was greater between the punch and die surface. The compressive strain intensity increased around the punch edge and there was convergence condensation between the punch and die edges, as shown in Figure 11. Therefore, the material behavior was characterized by large defects on the extrusion shrinkage, blank buckling, and extrusion die roll appearance.

## 4. Discussions

Comparing the observed results, it is clear that when the punch penetrated into the blank material, AISI-1045 steel in the SME process, it was able to flow deflate into the extrusion cavity and protrude on the opposite side. As the depth of the punch penetration increased, the material flowed through the die orifice, and the extruded material had defects that were visible [2, 5].

The microstructure of the material's grains' size was flat and very fine between the region of the punch and die edges, when the depth of the punch's penetration increased. Moreover, the compressive stress and strain were intense around the punch edge and there was convergence condensation between the punch and die edges. However, maybe there was weakness in the shock load. Thus, the sheet metal extrusion applications must be chosen carefully.

The FEM simulation error, according to the measured size, was smaller when verifying the FEM equation's error effect on the FEM results with measurement values. Namely, measuring the small size was a fractional error, and measuring the large size was a greater error in contrast.

## 5. Conclusion

From the present study, the conclusions can be summarized as follows.

The punch penetration on the blank material with extrusion ratio formed the extrusion parts. Moreover, when increasing the depth of the punch's penetration to over half of the blank's thickness, defects were then found that were not in the control material flow. However, increasing depths of punch penetration rendered a narrow region of highly localized plastic deformation that affected the material's gain and mechanical properties. The FEM results predicted formations that were identical in aspect direction to those found in the practical experimental results. Therefore, using the FEM simulation for metal forming investigations could aid understanding of the behavior, which would be helpful to decrease the cost, time and trial error, and so forth. If the FEM simulation was appropriately optimized, then an exact solution could be obtained.

## Figures and Tables

**Table 1:** FEM simulation conditions.

Simulation model	Axisymmetric model
Object type	Work piece: elastic-plastic Punch/die: rigid Blank holder: rigid Counterpunch: rigid Guide ring: rigid

Blank material	AISI-1045 *⌀*_*B*_ = 38, *t* = 5 mm *σ*_*B*_ = 530 MPa, *λ* = 26%

Punch penetration	*P*_depth_ = 1, 2 and 3

Punch	AISI-M4 *⌀*_*P*_ = 12.70 mm, *R*_*p*_ = 0.20

Die	AISI-M4 *⌀*_*d*_ = 10.00 mm, *R*_*d*_ = 0.20

Blank holder force	AISI-M4, *F*_*B*_ = 16 kN

Counter punch force	AISI M4, *F*_*C*_ = 500 N

Flow curve equation	$\overset{-}{\sigma }=850{\overset{-}{\varepsilon }}^{0.478}+385$

Fracture criterion equation	Oyane (constant *α* = 1, critical fracture value *C* = 0.157)

Friction coefficient	*µ* = 0.04
